# An analysis of the 6-h ultra-marathon race using a machine learning approach

**DOI:** 10.3389/fspor.2025.1577470

**Published:** 2025-09-22

**Authors:** Mabliny Thuany, Katja Weiss, David Valero, Elias Villiger, Marilia S. Andrade, Pantelis T. Nikolaidis, Volker Scheer, Claudio Andre Barbosa de Lira, Rodrigo Luiz Vancini, Ivan Cuk, Lorin Braschler, Thomas Rosemann, Beat Knechtle

**Affiliations:** ^1^ Department of Physical Education, State University of Para, Pará, Brazil; ^2^ Institute of Primary Care, University of Zurich, Zurich, Switzerland; ^3^ Ultra Sports Science Foundation, Pierre-Benite, France; ^4^ Department of Physiology, Federal University of São Paulo, São Paulo, Brazil; ^5^ School of Health and Caring Sciences, University of West Attica, Athens, Greece; ^6^ Faculty of Physical Education and Dance, Federal University of Goiás, Goiânia, Brazil; ^7^ MoveAgeLab, Physical Education Sport Center of Federal University of Espirito Santo, Vitoria, ES, Brazil; ^8^ Faculty of Sport and Physical Education, University of Belgrade, Belgrade, Serbia; ^9^ Faculty of Medicine, University of Bern, Bern, Switzerland; ^10^ Medbase St. Gallen Am Vadianplatz, St. Gallen, Switzerland

**Keywords:** ultra-endurance, extreme endurance, ultra-running, event location, origin, country

## Abstract

**Background:**

Ultra-marathon running popularity is increasing, with the 6-h run being the shortest time-limited ultra-marathon. Since very little is known regarding the country from which the fastest 6-h runners originate, the fastest age group, and where the fastest 6-h race courses are located, this study aims to close this gap.

**Methods:**

A machine learning model based on the XG Boost algorithm was built to predict running speed based on the athletés age, gender, country of origin, and the country where the race takes place. Model explainability tools were used to investigate how each independent variable would influence the predicted running speed. To assess the impact of individual performance against the other variables under study, a Mixed Effects Linear Model was also built.

**Results:**

A total of 117,882 race records from 51,018 unique runners from 65 countries participating in races held in 56 different countries were analyzed. Participation is spread across a wide range of countries, with a high correlation between the country of origin and the country of the event. Most runners originated from Germany, Italy, France, the USA, and Sweden, with Europe (Belgium, Russia, Spain, Poland, Romania, and Lithuania), being the fastest. Most athletes competed in Italy, Germany, France, the USA, and The Netherlands. The fastest average running speeds were also achieved in European countries (Russia, Belgium, Poland, Netherlands, Romania, Croatia, and Lithuania).

**Conclusions:**

For athletes competing in a 6-h ultramarathon, gender was the most important predictor, followed by the origin of the athlete, the age, and the race location. The 6-h running event seems to be dominated by European athletes regarding both participation and performance.

## Introduction

Ultra-marathon running is highly popular (1) and is typically held as time-limited (6 h, 12 h, 24 h, etc.) or distance-limited (50 km, 100 km, 50 miles, 100 miles, etc.) races (2). The 6-h ultra-marathon is the shortest time-limited ultra-marathon, where amateur and non-elite athletes have enough time to complete a marathon. Different studies have been conducted on this race format to investigate biomechanical aspects such as stride frequency (3), changes in running mechanics (4), and running kinematics (5). Further studies investigated physiological aspects such as the influence on cardiorespiratory response (6, 7) or heart rate variability. Another topic was neurological aspects such as the effect of 6-h running on brain activity and cognitive performance (8), neuromuscular responses, or postural control after a 6-h ultra-marathon (8).

Despite available data regarding biomechanics and physiological aspects, a broader analysis of the profile of these competitors and the race courses is lacking. This information is useful to guide future endurance events, especially considering that over the last few years, the number of ultra-marathons has grown worldwide (9), which can also increase the possibilities for participation in these events for athletes who usually need to travel long distances to attend in the past. Available information regarding participation and performance trends of these runners until 2010, found that Europeans were the most numerous and achieved the best top ten performances (10). However, it could not represent the current scenario. Regarding the nationality/origin of ultra-marathoners, the time frame of the considered data and the statistical approach can lead to very different results. A recent study investigating the top ten 100-km ultra-marathoners by nationality showed that Japanese runners were the fastest worldwide (11). This study used data from 112,283 athletes who completed a 100-km ultra-marathon worldwide between 1998 and 2011 and the statistical approach was single- and multi-level regression analyses. A more recent study investigating data from 150,710 athletes who finished a 100-km ultra-marathon between 1959 and 2016 showed that runners from Russia were the fastest (12). This study used, however, both a linear regression and a truncated regression. Most probably, the number of athletes (i.e., the considered time frame) and the statistical approach seemed to lead to different results.

In the same direction, the age of peak performance has been previously investigated for over 10 years (10). However, limited information exists about sex and age-related differences in participation and performance in 6-h races. A previous study investigating sex differences for time-limited events from 1990 to 2020 (13), showed a reduction in the gap between men and women competing in a 6-h ultramarathon. Similarly, sex and age were significantly associated with speed (13), but more detailed information about the most popular age groups is lacking. Knowledge about the age of participants in these races can be important for health professionals working with them to develop age-specific prevention and physical training programs.

Since we also do not know where the fastest 6-h races were held, our primary aim is to investigate the origin of the fastest 6-h ultra-marathoners and the location where the fastest races were held using a machine-learning model. This approach has been successfully used in studies investigating triathlon races (14) and other ultra-marathon running races (14–16). Based on previous research, we hypothesized that Europeans were the fastest runners and that the fastest races would be held in European countries. A secondary aim of the present study was to examine the variation of participation and performance by age group and sex. Based upon recent findings, we hypothesized to find an age of peak performance at ∼30–35 years. Furthermore, we used a different statistical analysis with a machine learning approach.

## Methods

### Ethical approval

This study was approved by the Institutional Review Board of Kanton St. Gallen, Switzerland, with a waiver of the requirement for informed consent of the participants as the study involved the analysis of publicly available data (EKSG 01/06/2010). The study was conducted following recognized ethical standards according to the Declaration of Helsinki adopted in 1964 and revised in 2013.

### Data analysis

#### Study design and data processing

This is a descriptive observational study developed with 6-h ultra-marathoners from all over the world. Race data was downloaded (https://statistik.d-u-v.org) using a Python script. Each race record included the athletés name, age group and gender, country of origin, race location and year, race length (duration), and the athlete's race time. To reduce noise and maintain the statistical significance of the results, race records from athlete countries with fewer than 10 records were removed, and the same procedure was applied to records from event countries with fewer than 10 records. While these measures will allow for a reduction of noise and a more straightforward interpretation of the results, some countries/runners will have been unwittingly omitted.

### Outcome variable

Performance was quantified in terms of race average speed (in km/h). This decision was based on the lack of information regarding the personal best time and previous literature (9, 17).

### Independent variables

The following variables were used as predictors or inputs to the model: gender, age group, athlete country, and race location*.* The gender variable is encoded as female = 0 and male = 1. The age group numerically encoded in 5-year groups except group 18, which represents runners of less than 20 years, and group 75, which represents 75 years and older. The athlete's country and race location variables are encoded based on their position in the respective **rankings**, with the fastest average running speeds at the top.

### Statistical analysis

Descriptive statistics were computed using percentages, means, and standard deviations. A matrix of Pearson correlation was calculated to verify the relationship between predictors, especially focusing on the athlete's country and race location variables. Following, we used the XG Boost algorithm to verify the association between gender, age group, athlete country, and race location with performance. The sample used to build, evaluate, and interpret the XG Boost regression model consists of **117,882 unique race records** from 51,018 unique runners from 65 different countries of origin, with races taking place in 56 different event countries. Two evaluation metrics, Mean Absolute Error (MAE) and *R*^2^, are calculated along with the model's relative features’ importance. Interpretation tools based on Partial dependence plots (PDP) calculations were used to visualize the model. Descriptive stats of average speeds are shown alongside, for an easy analysis. The combo of prediction distribution/target/group size plots shows the range of predictions of the model as boxplots for different values of each predictor. A hold-out evaluation strategy was used to tune the model by iterative training and evaluating different models with different test splits and different numbers of estimators/learn rates. After several iterations and tests, the optimal model parameters and accuracy scores were presented. All computation and analysis were done using a Jupter notebook (Google Colab, USA) and Python and associated libraries (pandas, numpy, xgboost, pdpbox, sklearn, matplotlib, and sns). A confidence interval of 95% was adopted.

## Results

After all necessary processing, the final 6-h race sample consists of **117,882 race records** from **51,018 unique runners from 65 different countries** participating in **races held in 56 different countries**. Table 1 summarizes the race records by the 65 countries of origin of the athletes. Most runners originated from Germany (16.9%), Italy (16.6%), France (11.2%), the USA (10.6%), and Sweden (5.0%). The fastest average running speeds over 10 km/h are achieved by several European countries (Belgium, Russia, Spain, Poland, Romania, Lithuania) with a peak of over 12 km/h of Israeli, which with only two unique runners, is more likely related to individual performance.

**Table 1:** *Athlete country* by the ranking of race speed, race records, and runners.

Ranking	Country	Mean running speed (km/h)	std	Minimum running speed (km/h	Maximum running speed (km/h)	Race records	Unique runners
**0**	GER	9.46	1.34	6.19	14.87	19,933	6,908
**1**	ITA	9.24	1.25	7.50	15.07	19,569	5,527
**2**	FRA	9.57	1.33	6.76	14.85	13,194	6,481
**3**	USA	8.83	1.11	3.76	20.64	12,543	8,153
**4**	SWE	9.49	1.31	7.50	15.26	5,940	2,482
**5**	TPE	8.93	1.04	2.04	13.55	5,190	2,550
**6**	AUT	9.76	1.35	7.50	14.25	4,060	1,433
**7**	NED	9.99	1.33	6.88	14.54	3,996	994
**8**	BEL	10.58	1.62	7.50	15.30	3,222	862
**9**	HUN	9.87	1.33	6.16	15.44	3,208	1,488
**10**	RUS	10.48	1.56	5.52	15.54	2,557	982
**11**	AUS	9.46	1.36	7.50	17.29	2,398	1,356
**12**	DEN	9.89	1.24	5.55	14.53	2,385	1,120
**13**	CAN	9.12	1.13	7.50	13.60	2,172	895
**14**	NOR	9.72	1.43	4.15	14.80	2,013	806
**15**	JPN	9.67	1.42	7.50	15.10	1,878	1,097
**16**	GBR	9.28	1.42	7.50	16.20	1,817	1,081
**17**	FIN	9.43	1.25	7.51	14.68	1,552	742
**18**	ESP	10.05	1.58	7.50	15.08	1,503	767
**19**	CZE	9.62	1.29	7.50	14.45	778	368
**20**	ARG	9.40	1.25	7.50	14.38	754	483
**21**	SLO	9.71	1.47	7.48	13.97	700	341
**22**	BRA	9.35	1.24	7.00	14.76	558	450
**23**	CHN	9.39	1.30	7.60	14.54	518	445
**24**	SRB	9.36	1.34	7.00	14.82	477	204
**25**	POL	10.55	1.74	7.50	15.83	465	324
**26**	SUI	9.78	1.44	7.50	14.07	436	203
**27**	GRE	9.43	1.24	7.50	14.60	416	311
**28**	SVK	9.73	1.26	7.50	14.07	391	177
**29**	URU	9.37	1.18	7.52	14.13	326	224
**30**	ROU	10.04	1.49	7.51	14.48	285	170
**31**	BUL	9.32	1.08	7.56	12.42	237	159
**32**	UKR	9.81	1.46	7.50	14.53	223	157
**33**	HKG	9.21	1.17	7.50	12.49	205	153
**34**	CRO	9.85	1.20	7.50	13.42	193	141
**35**	NZL	9.43	1.15	7.50	12.27	190	127
**36**	BLR	9.77	1.46	7.50	13.69	168	77
**37**	IRL	9.75	1.38	7.54	13.03	162	105
**38**	LTU	10.47	1.52	7.54	16.42	135	73
**39**	EST	9.53	1.26	7.58	13.51	107	68
**40**	IND	9.31	1.41	7.51	13.03	105	86
**41**	RSA	9.41	1.61	7.51	13.87	83	69
**42**	LAT	9.95	1.36	7.51	12.63	82	51
**43**	MDA	9.36	1.25	7.54	14.05	79	37
**44**	BIH	9.58	1.51	7.56	13.70	69	37
**45**	KOR	9.41	1.14	7.50	12.88	67	62
**46**	POR	9.73	1.38	7.53	12.28	61	40
**47**	MAS	8.56	0.77	7.50	11.05	49	41
**48**	PHI	8.73	0.95	7.50	11.08	46	8
**49**	SMR	8.79	0.85	7.50	10.24	46	26
**50**	LUX	9.43	1.25	7.65	12.64	42	21
**51**	KAZ	9.48	1.44	7.50	12.02	40	27
**52**	MEX	9.07	1.23	7.61	11.53	38	27
**53**	ISR	10.65	0.66	9.23	12.11	32	2
**54**	MGL	9.36	1.17	7.50	11.46	27	27
**55**	TUR	9.90	1.38	7.54	12.07	23	11
**56**	ISL	9.92	0.82	8.72	11.42	22	3
**57**	PER	8.24	0.62	7.53	9.87	22	14
**58**	TUN	12.28	1.51	7.83	14.30	18	2
**59**	ALB	9.30	1.15	7.50	11.30	16	6
**60**	ECU	9.36	0.95	7.77	11.07	15	13
**61**	ALG	9.43	0.71	8.60	10.67	13	10
**62**	MKD	9.14	1.16	8.00	11.19	13	5
**63**	SGP	9.31	0.99	7.65	10.84	11	8

Min (race speed minimum value); max (race speed maximum value); race speed in km/h; and sd (standard deviation).

Table 2 summarizes the events of 56 different countries. Most athletes competed in Italy, Germany, France, the USA, and the Netherlands. The fastest average speeds are achieved also in European countries such as Russia, Belgium, Poland, the Netherlands, Romania, Croatia, and Lithuania.

**Table 2:** *Event country* ranking.

Ranking	Event Country	Mean running speed (km/h)	std	Minimum running speed (km/h	Maximum running speed (km/h)	Race records	Unique runners
**1**	ITA	9.25	1.26	7.50	15.07	19,756	5,705
**2**	GER	9.44	1.34	6.19	15.01	18,933	7,057
**3**	FRA	9.62	1.34	6.76	15.30	14,258	7,352
**4**	USA	8.83	1.10	3.76	20.64	12,562	8,221
**5**	NED	10.13	1.45	6.88	15.36	6,431	1,979
**6**	SWE	9.49	1.31	7.50	15.26	5,961	2,560
**7**	TPE	9.00	1.09	2.04	13.55	5,597	2,779
**8**	AUT	9.77	1.36	7.50	14.91	4,002	1,554
**9**	HUN	9.82	1.30	6.16	15.44	3,111	1,504
**10**	RUS	10.45	1.53	5.52	15.54	2,540	999
**11**	AUS	9.45	1.37	7.50	17.29	2,510	1,465
**12**	DEN	9.87	1.24	5.55	14.53	2,321	1,166
**13**	CAN	9.12	1.13	7.50	13.53	2,152	914
**14**	NOR	9.69	1.41	4.15	14.80	2,026	904
**15**	BEL	10.47	1.58	7.50	15.01	2,012	903
**16**	ESP	10.03	1.54	7.50	15.21	1,625	910
**17**	FIN	9.41	1.23	7.51	14.68	1,623	849
**18**	GBR	9.20	1.41	7.50	16.42	1,605	1,005
**19**	JPN	9.59	1.43	7.50	15.10	1,581	1,016
**20**	CZE	9.67	1.29	7.50	15.08	827	551
**21**	ARG	9.36	1.21	7.50	14.38	776	531
**22**	SLO	9.70	1.44	7.50	14.49	662	346
**23**	BRA	9.28	1.19	7.00	13.65	517	426
**24**	SRB	9.43	1.38	7.00	14.82	507	267
**25**	CHN	9.38	1.32	7.60	14.54	474	439
**26**	GRE	9.36	1.16	7.50	14.16	385	303
**27**	SVK	9.72	1.29	7.50	14.07	374	223
**28**	SUI	9.49	1.37	7.50	14.87	362	264
**29**	POL	10.70	1.83	7.50	15.83	328	262
**30**	URU	9.41	1.29	7.52	14.13	266	212
**31**	ROU	10.04	1.52	7.54	14.26	224	161
**32**	BUL	9.32	1.13	7.56	13.09	220	162
**33**	HKG	9.24	1.23	7.50	13.01	157	138
**34**	CRO	10.08	1.43	7.50	13.80	127	122
**35**	NZL	9.56	1.18	7.53	12.27	126	97
**36**	UKR	9.55	1.47	7.50	13.69	114	104
**37**	BLR	9.56	1.33	7.50	12.61	107	57
**38**	EST	9.79	1.34	7.58	12.78	104	80
**39**	IRL	9.52	1.53	7.54	14.69	83	71
**40**	LTU	10.17	1.33	7.54	13.40	75	57
**41**	RSA	9.30	1.62	7.51	13.87	66	56
**42**	IND	8.79	1.10	7.51	11.47	64	62
**43**	KOR	9.22	1.04	7.50	12.88	55	54
**44**	MAS	8.47	0.67	7.50	10.12	44	40
**45**	LAT	9.86	1.54	7.51	12.50	39	29
**46**	MDA	9.20	1.33	7.54	14.05	35	33
**47**	KAZ	8.96	1.20	7.50	12.02	29	25
**48**	LUX	9.46	1.46	7.65	12.68	22	16
**49**	MON	8.90	1.08	7.59	11.38	18	18
**50**	POR	9.47	1.40	7.72	12.28	16	13
**51**	BIH	9.06	1.32	7.52	11.59	14	14
**52**	MEX	8.17	0.91	7.67	10.17	13	12
**53**	PHI	8.61	0.99	7.50	10.83	13	11
**54**	ISL	9.97	0.80	8.72	11.22	12	11
**55**	MGL	9.16	1.11	7.50	10.68	11	11
**56**	ECU	9.47	1.00	7.77	11.07	10	10

Min (race speed minimum value); max (race speed maximum value); race speed in km/h; and sd (standard deviation).

**Figure 1:**
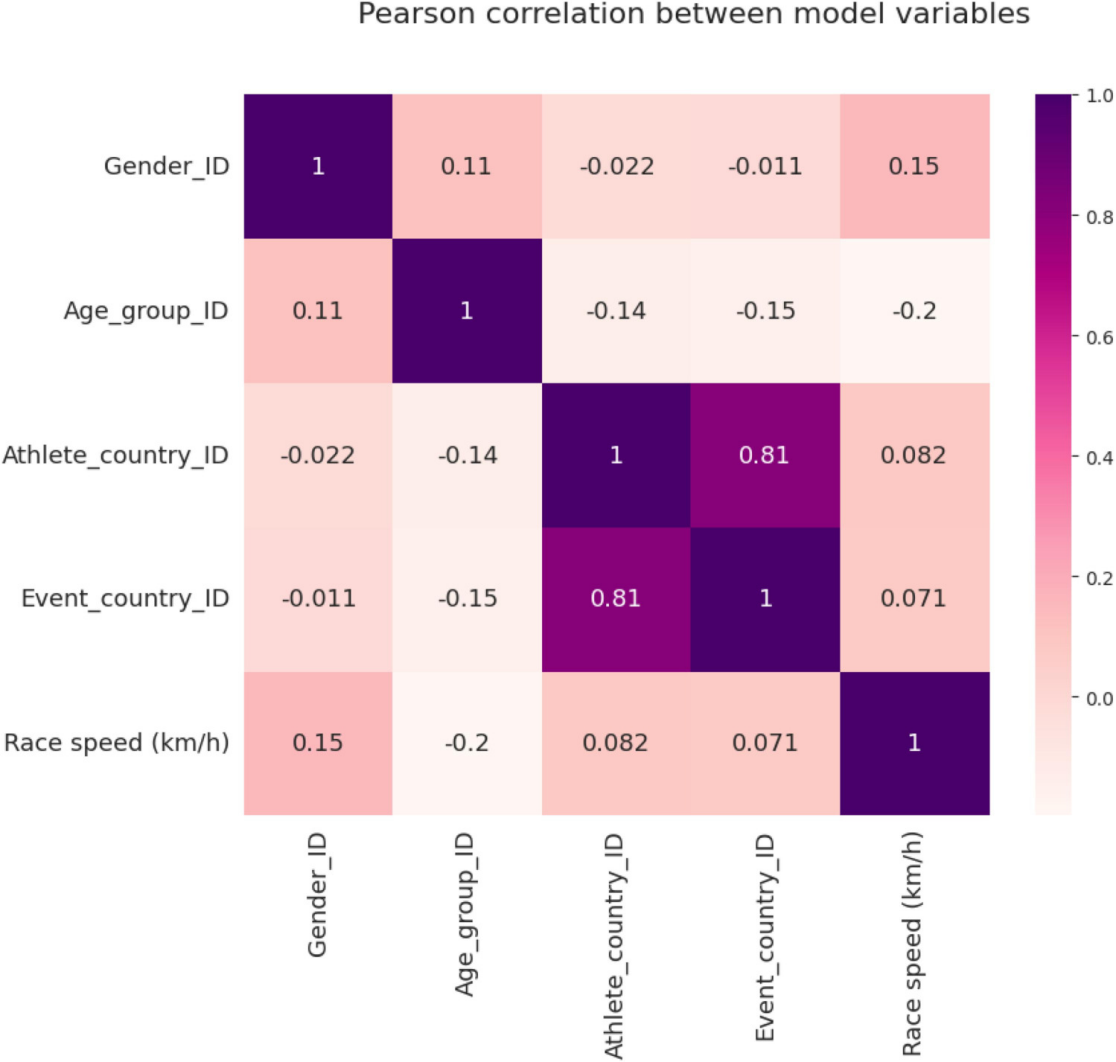
Correlation matrix between model variables.

A correlation matrix between predictors and target has been plotted (Figure 1). There is a high correlation between the country of the athlete (origin of the athletes) and the country where the event was held (Pearson *r* = 0.81).

**Figure 2:**
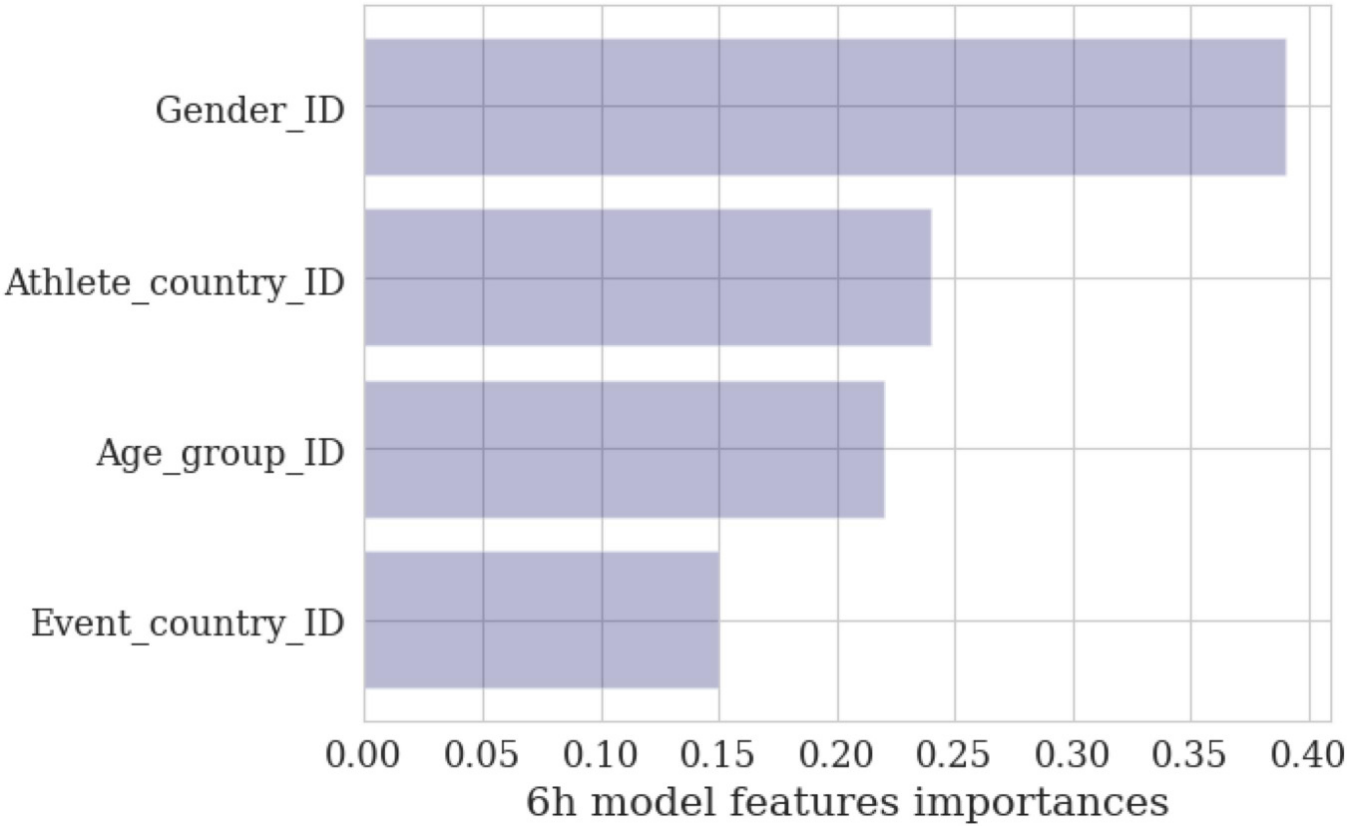
The model features relative importance.

The association between the predictors and the speed is presented in Figure 2. The model rates gender (0.39) as the most important predictor (based on data entropy reduction), followed by athlete country (0.24), age group (0.22), and event country (0.15). The “optimal” model [sample size 117,882, XG Boost trees 200, learning rate 0.5,] obtained ***R*^2^** **=** **0.21 and MAE (km/h) 0.97**, which indicates that about 21% of speed variability is explained by the predicting variables in the model. The value of *R*^2^ = 0.21 is quite low for any predictive purposes, suggesting that additional predicting variables should be added to the model to improve its predictive power.

**Figure 3:**
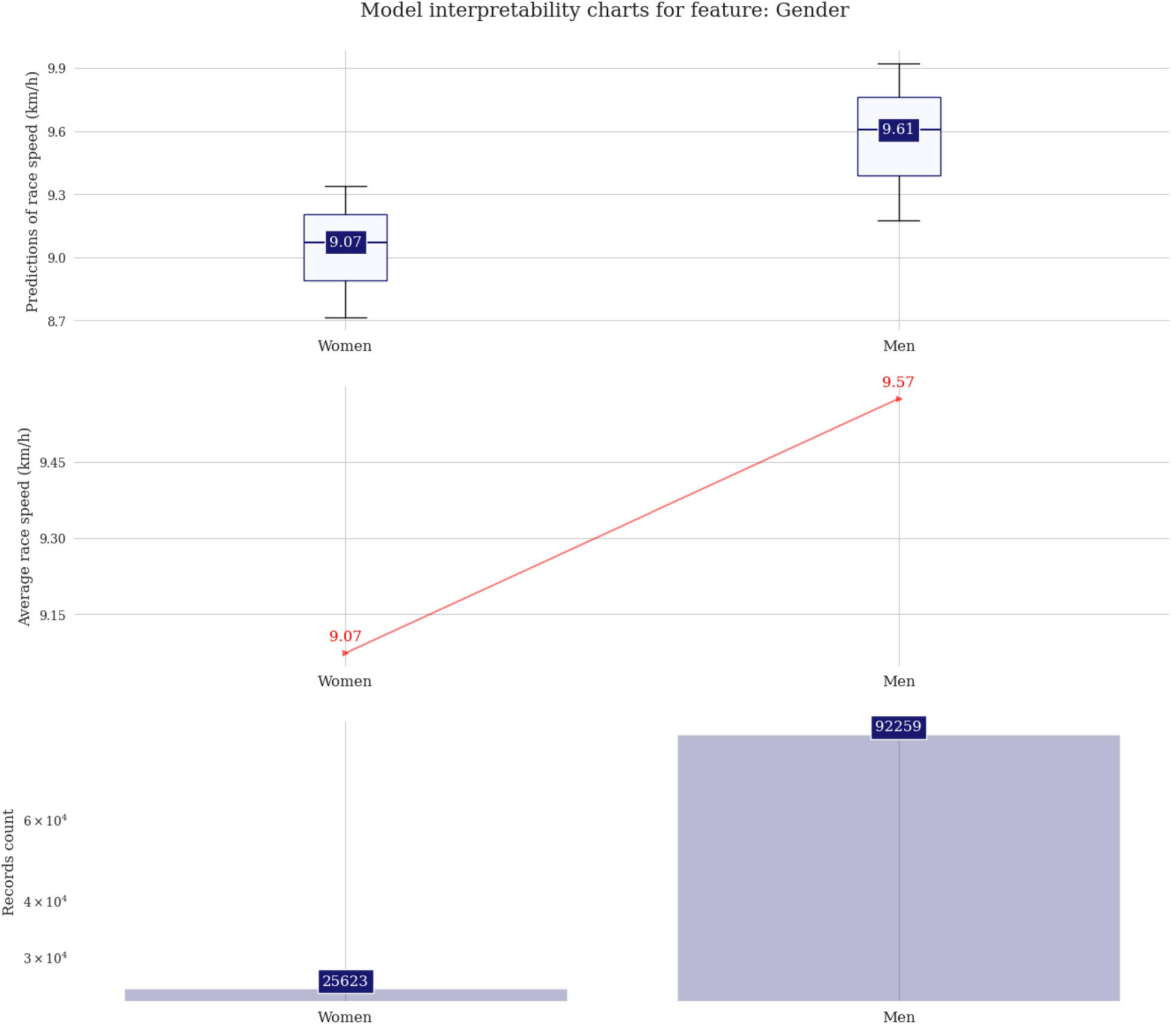
Prediction distributions and target plots for gender.

**Figure 4:**
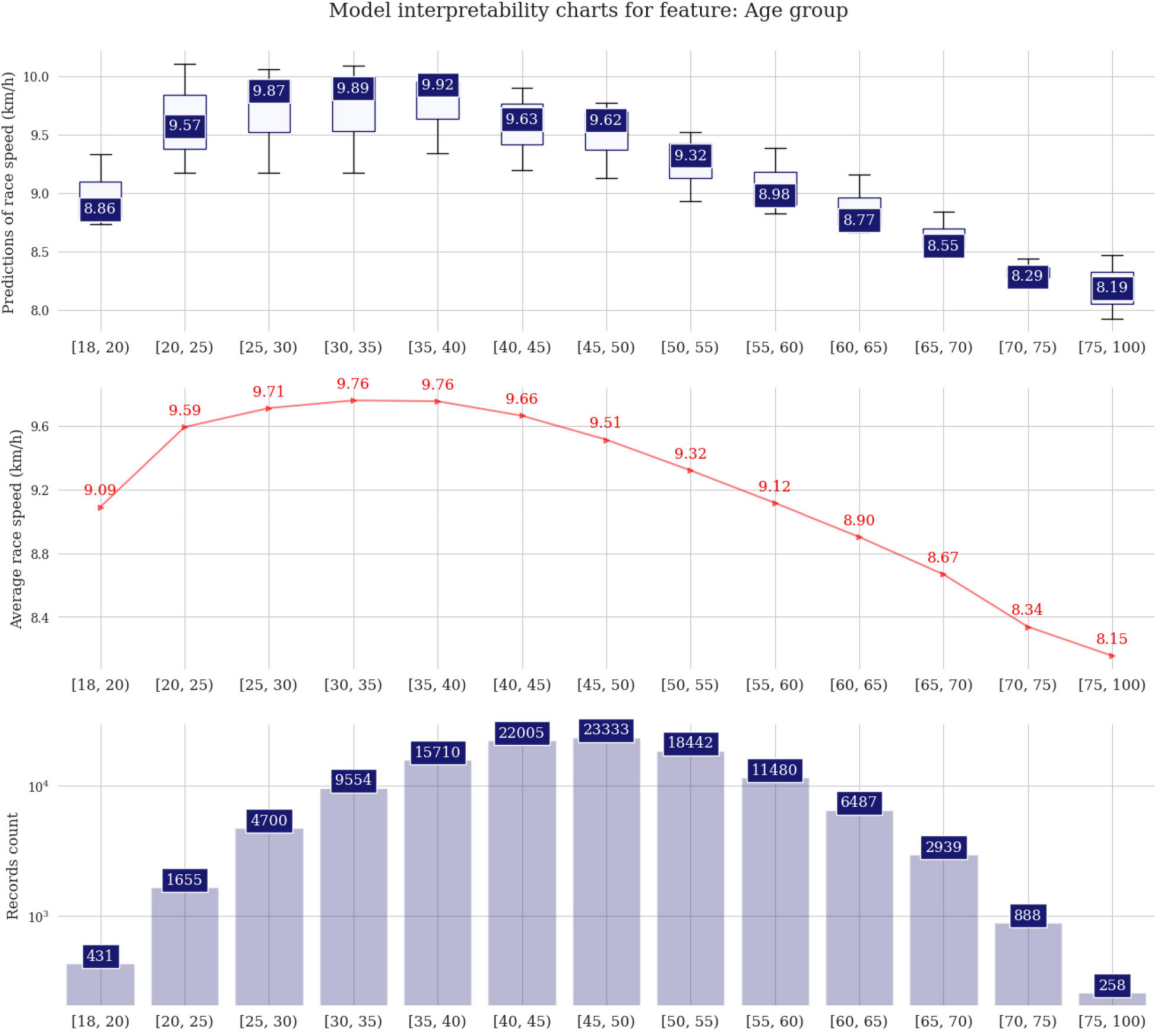
Prediction distributions and target plots for age group.

**Figure 5:**
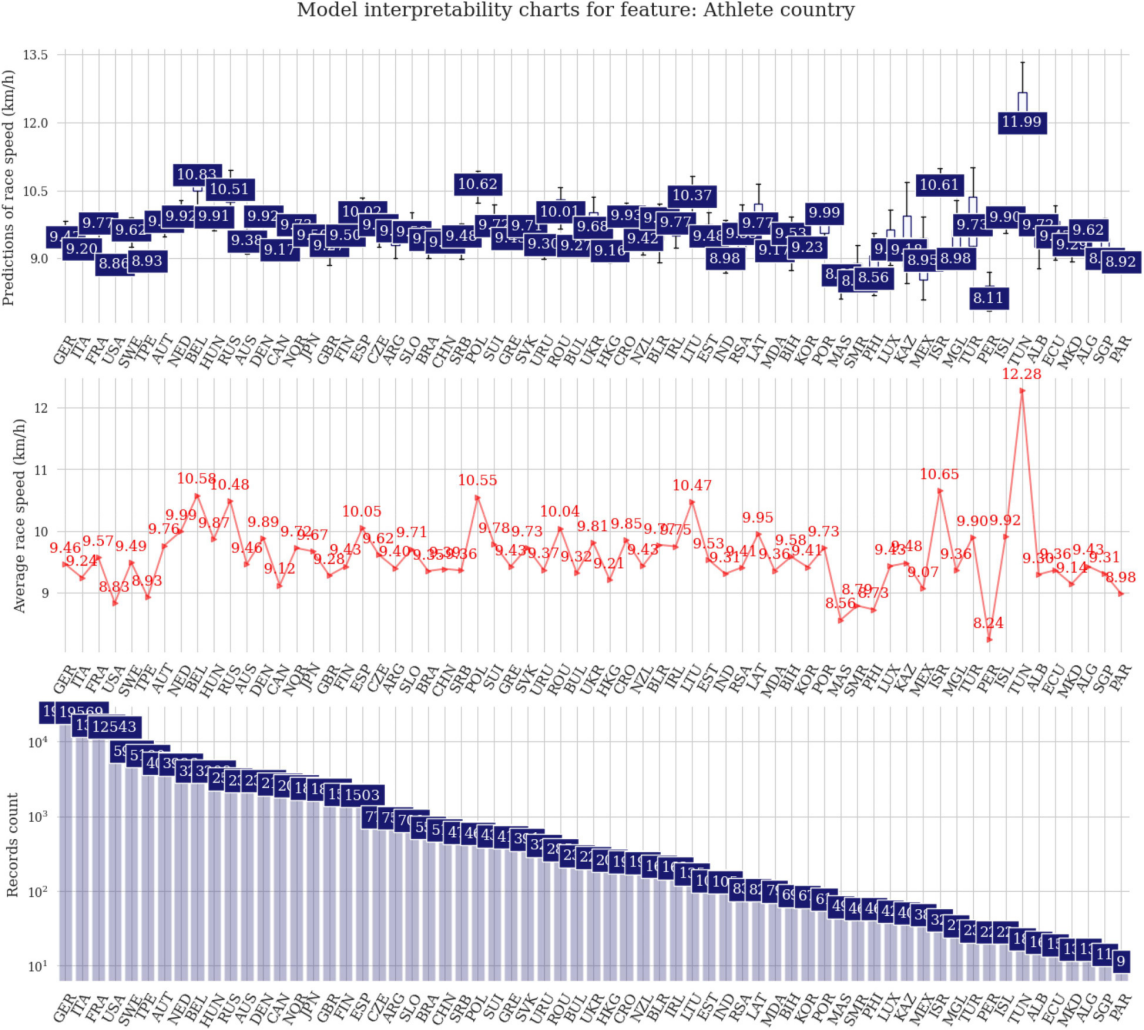
Prediction distributions and target plots for origin of the athlete.

The difference among male and female predictions is about 0.5 km/h on average (Figure 3). The fastest runners are in age groups 25, 30, or 35 years, with the first two groups showing almost identical distribution boxes. Regarding participation, the age group 45 years is the highest (Figure 4). In the athlete country charts, a distinctive peak can be seen for Tunisia, with predicted running speeds over 12 km/h but with only two unique runners in the sample, followed by Belgium, Russia, Poland, Lithuania, and Israel with predictions of ∼10.5 km/h (Figure 5).

**Figure 6:**
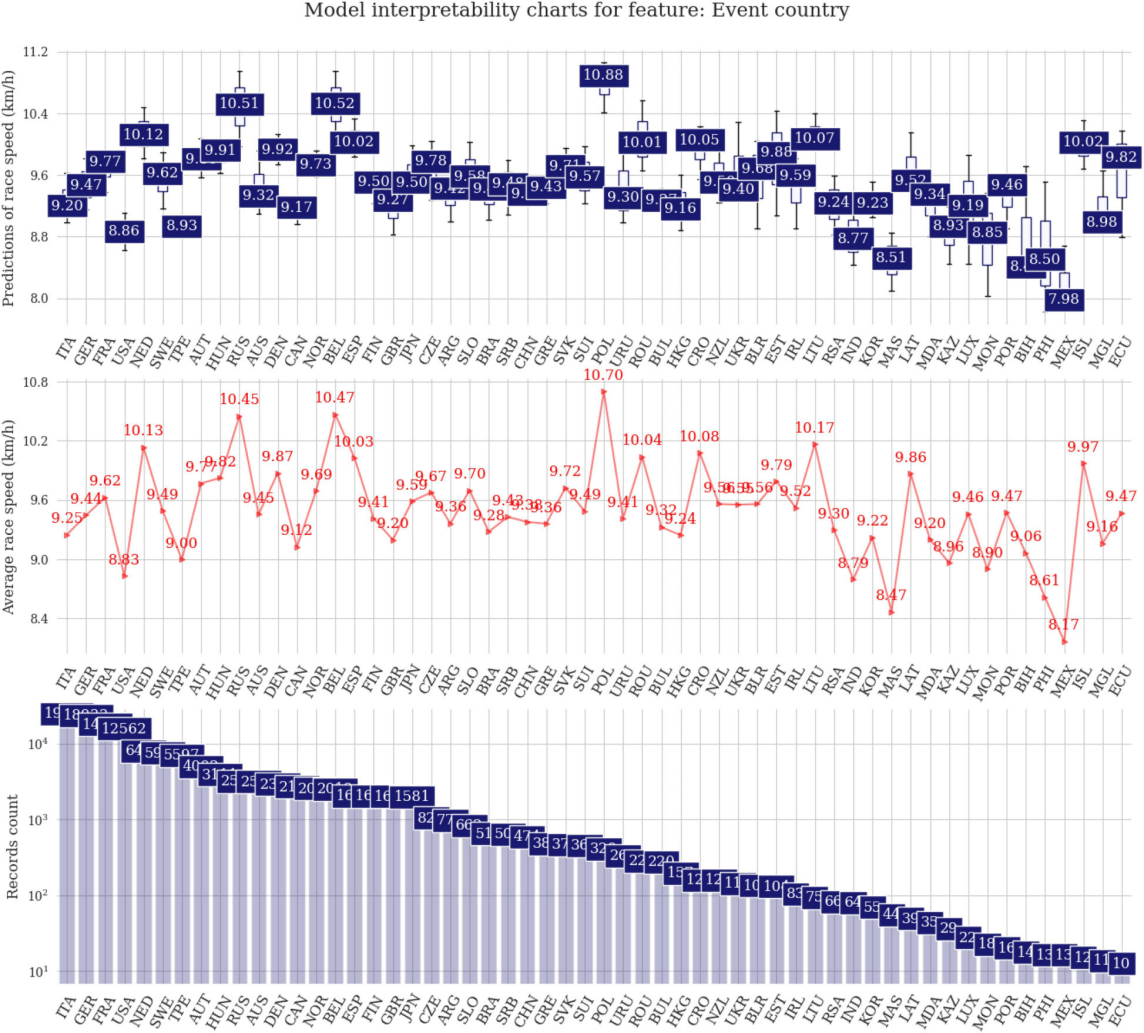
Prediction distributions and target plots value plots for the country where the events were held.

In the event country charts, it is possible to identify a similar lineup, including Belgium, Russia, and Poland, pointing to the correlation between country of origin and country of event. Some other European countries, including the Netherlands, Spain, Romania, or Croatia can be added to the countries holding 6-h races with average running speeds over 10 km/h (Figure 6).

## Discussion

In this study, we investigated multiple variables of the 6-h ultra-marathon event, exploring the origin of the fastest runners, dissecting age group differentials, scrutinizing gender variations, and examining the geographical distribution of the fastest race courses. Our findings showed that i. gender was the most important predictor for performance, followed by the origin of the athlete, the age, and the race location, ii. most runners originated from Germany, Italy, France, the USA, and Sweden, iii. the fastest average running speeds are achieved by European athletes competing in Europe. These results are aligned with our hypothesis, that Europeans exhibit a superior performance and hosted the fastest 6-h races. Furthermore, our secondary hypothesis was that male athletes demonstrated higher running speeds, challenging the notion that younger participants are consistently the fastest in this endurance domain. By exploring these variables, we aimed to provide nuanced insights into the multifaceted determinants of success in 6-h ultra-marathons.

### The fastest 6-h runners originated from European countries

We confirmed recent findings indicating that Europe has the highest proportion of athletes, the best performances, and the fastest race courses (10). These results are aligned with the high correlation between the country of the athlete (origin of the athlete) and the country where the event was held and should be analyzed with caution. These results can be biased by the number of race events held between continents. Data covering race events globally, indicates a higher portion of events in Europe (622), compared to North America (348), Asia (208), Africa (55), South America (38), and Australia (35). Specifically for the present research, in absolute terms, 19,756 (16.8%) were held in Italy, 18,933 (16.1%) in Germany, and 14,258 (12.1%) in France. These results can be related to the advantages of competing at home, with reduced costs of accommodation, traveling, and time management, as well as adaptation to weather conditions and race characteristics. Despite a previous idea that logistical aspects influence race events participation, the magnitude of the influence on endurance performance needs to be investigated, similar to the association between performance improvement and re-participation in running events, which could partially explain the present findings, and can also be related to the findings related to the fastest race courses.

Previous studies tried to understand the pattern of participation and performance among race distances, with few data available regarding race courses. A previous study on ultra-marathoners competing in 100-mile events found that the fastest male runners were from Africa, while the fastest female runners were from Europe and Africa (18). Following these results, women from Sweden, Hungary, and Russia performed best in the top three, top ten, and top 100, while men from Brazil, Russia, and Lithuania were the fastest (18). Despite the authors using a macro-to-micro analysis, studying world regions and countries, no information was presented by the fastest race course.

A previous study using machine learning models to identify the fastest racecourse among elite athletes competing in triathlon showed that the level of the competition was the most important characteristic to predict performance, compared to the place where the event takes place (15). For runners competing in an event held in the USA, performance and participation were dominated by athletes from the USA, whereas the contrary was shown for those competing in events held in Europe (16). These results were similar to those of athletes competing in the “100 km Lauf Biel” in Switzerland the most traditional 100 km ultra-marathon worldwide, with the results showing a higher portion of participants from European countries, which could be explained by the large distance between the USA and Europe, and by the fact that US-American ultra-marathoners preferably compete in ultra-marathons held in the USA (11).

These results are aligned with our findings, which suggest that the place of competing influences performance. Although we did not test for this association, some factors can explain these results. For example, ultra-marathon events usually happen under several geographical and weather conditions, which can be positively managed by runners who know these characteristics (14). An interesting aspect is the fact that non-elite (recreational) runners can complete a standard marathon within 6 h. Regarding marathons, we know that East African runners dominate marathon races (19). Interestingly, no East African nation is among the fastest in running a 6-h ultra-marathon. The most likely explanation is that some of them, like Kenyans, prefer to compete in running races such as large city marathons for financial reasons (19). Despite this information being potentially useful for athletes and coaches aiming to achieve personal best performances, the results should be interpreted with caution. Detailed information about the race courses—such as elevation profile, altitude, surface type, climate conditions, and support infrastructure (e.g., aid stations, pacing availability)—is not available, which may significantly influence performance outcomes and limit the generalizability of the findings.

### Men and athletes in the age group 30–34 years were the fastest

Despite the interesting possibility that women outperform men in ultra-endurance sports disciplines due to the capacity to use fatty acids and preserve carbohydrates during prolonged exercise, presents a more even pacing strategy, and less fatigue following endurance running exercise present study indicates that men are faster (20). Despite previous findings reporting a gap reduction for those competing gin 6-h events, men continue to be faster compared to women, regardless of the competitive level (e.g., elite, or non-elite) (21). In this sense, these findings follow previous studies in which men presented higher performance compared to women (9, 22).

Factors that explain sex differences include both biological and sociocultural characteristics since men present higher values for the maximal oxygen uptake (∼10% higher VO_2_max), been considered the most important indicator of endurance activities (20). These differences are mainly due to the men have due to greater muscle mass, higher hemoglobin, and larger hearts and lungs (20). In addition to biological factors, gender disparities in access to opportunities, funding, financial incentives, and social support from peers and governing bodies may help explain the lower participation of women in competitive sports (23). Moreover, persistent stereotypes and traditional views of women's roles in society continue to serve as significant barriers (24).

Conversely, in the context of longer-distance ultra-marathon races, where the demands on aerobic and psychological endurance are heightened, there is a suggestive trend that athletes aged 45 years or over might exhibit enhanced performance. This nuanced observation underscores the intricate interplay between age, physiological demands, and race duration, contributing to a more comprehensive understanding of how different age groups navigate the varied challenges posed by distinct types of running events. For age groups, these results differ from previous publications. In 50 km ultra-marathoners, the peak performance varies based on the statistical approach, and it can be 41 years of age or declining (25). Regarding participation trends, female runners of a mean age of about 43 and 46 years showed the highest performance in women and men, respectively (25). The different results can be related to the methodological decision and the statistical approach used. For example, the studies adopted different age groups, which can influence the summary of findings.

In addition, the higher performance among athletes aged between 30 and 34 years can be related to the natural aging process experienced by those with older ages. A previous study about neuromuscular characteristics in marathoners of different age groups showed an inverse and moderate correlation of the indices of muscle velocity and relative power with age, with groups older than 60 years presenting the lowest scores (26). Also, the authors report a moderate correlation between muscle strength and power with race time. Despite the focus on marathons, a previous study showed an association between knee extensor strength and race time among ultra-marathoners competing in mountain events (27). It should be highlighted that the age of peak performance in 6 h running was younger compared to longer time-limited ultra-marathon races (28). The actual age of peak performance in 6-h running can be considered closer to the age of peak physiological parameters related to performance such as maximal oxygen uptake and muscle strength, suggesting that the performance depended partially on physiological characteristics (29). Importantly, given the aging demographic trend, there appears to be a growing significance in focusing efforts towards enhancing athletic longevity, especially considering the increasing number of individuals participating in competitive sports at more advanced ages (30).

In this sense, our findings showed that most of the participants are aged between 45 and 49 years. These results agree with previous analysis of other time-limited events (12 h, 24 h), in which a trend of aging was shown, with a mean age of 45.62 ± 10.80 years (13). Besides the importance of understanding the ultramarathoners profile, these findings can contribute to the broad field of physical activity, highlighting endurance activities as a strategy to promote health among the general population. Another important point is the trend of aging among the population. Recent statistics show that the number of older people will reach over 1.5 billion in 2050 (31), which also calls for researchers to consider initiatives among this group. Following the results of the present study, and even though training and completing a long-distance running event such as ultra-marathons are different from starting a physical activity practice, the main findings of the present study can provide some insight for future interventions. We found that runners from countries with a higher number of events were those with a higher participation rate. Nevertheless, the existence of so many master athletes should draw the attention of medical and sports science personnel (e.g., coaches and fitness trainers) to develop age-specific exercise programs. Such age-tailored programs should consider the decline in cardiorespiratory fitness but also the more pronounced decline in muscular fitness (32).

In addition to this, the Athletic Career Transition model (33) could provide interesting insights regarding the natural transition between stages of the athletic career. The Athletic Career Transition model postulates different stages that the athlete experiences, from initiation to the post-career transition. In this sense, runners of different age groups could experience different motivations and levels of engagement. These differences can be related to changes in lifestyle, motivation to engage in competitive activities, among others. Understanding this shift between career stages reinforces the importance of developing age-specific training and support strategies that maintain running training.

### Limitations and strengths

The results summarized the observations across the descriptive charts (i.e., target plots) and the model interpretability charts (i.e., PDP and prediction plots). An important limitation is the XGBoost's susceptibility to overfitting, particularly when modeling complex, non-linear relationships in subgroups with limited representation. To reduce this bias, some countries with small sample sizes may have been excluded from the analysis. We also excluded race records where first name, last name, country of origin, or age was missing. Another important aspect to be considered was the reliability of the data. Since we were using secondary data from the official results of the events, we cannot guarantee the accuracy of the information. In addition, athletes could change their country of residence/nationality over the years, which was not considered in the present study. Similarly, qualitative information regarding the event location was not available. This was an important limitation because it impaired the generalization of the findings regarding the environmental characteristics that had a positive impact on athletes’ performance. The “optimal” model obtained a relatively low ***R*^2^**, indicating an **existing but weak effect** of the predicting variables in the model output, suggesting that additional predicting variables should be added to the model to improve its predictive power (such as altitude, temperature, training load, footwear type). A further limitation was that aspects such as weather (34), training culture (35), and environmental conditions (34)– which could have an impact on performance—were not considered. Most of the races included in the study are held in Europe, which may skew the results toward European dominance (36). Some countries, like Tunisia and Israel, have very few race records but show unusually high performances. This is most likely due to a selection bias of the best runners of these countries and these outliers should not lead to an over-interpretation of the findings. On the other hand, of the present study was its novel methodological approach since it was the first time that a machine learning model was used to predict 6-h running performance based on age, gender, country of origin, and event country. Furthermore, while the current study did not originally adopt a formal theoretical framework, our findings align with the Ecological Dynamics approach (37), that consider that the behavior (i.e., performance) emerge from the interaction between environmental factors (e.g., geographical distribution of events, climate, logistical accessibility), individual characteristics (e.g., age, sex, physiological capacities, experience), and event-related aspects (e.g., course profile, competition level), which provide practical information for professionals working with ultramarathon runners to set optimal performance goals depending on the event country.

## Conclusion

The present study aimed to provide a comprehensive examination of the factors influencing participants' performance in a 6-h ultra-marathon through the application of a predictive XG Boost model. By leveraging machine learning techniques, the study seeks to identify key predictors and environmental conditions, that significantly impact athletes' endurance and overall race outcomes. The analysis contributes to the understanding of ultra-marathon dynamics and holds practical implications for athletes, coaches, and event organizers by offering insights into optimizing training strategies and improving race-day performance in this challenging and demanding sporting context. In summary, the regression model rated gender as the most important predictor, followed by the origin of the athlete, the age, and the race location. The 6-h running event seems to be dominated by European athletes regarding both participation and performance. Although most runners were recorded in the age group 45-49 years, the fastest running speeds were achieved by athletes in the age group 30-34 years before age groups 25-29 and 35-39 years.

## Data Availability

The raw data supporting the conclusions of this article will be made available by the authors, without undue reservation.

## References

[1] RunRepeat. The state of ultra running 2020 (2024). Available online at: https://runrepeat.com/state-of-ultra-running (Accessed September 24, 2021).

[2] Scheer V Basset P Giovanelli N Vernillo G Millet GP Costa RJS. Defining off-road running: a position statement from the ultra sports science foundation. Int J Sports Med. (2020) 41:275–84. 10.1055/a-1096-0980 32059243

[3] Vernillo G Doucende G Cassirame J Mourot L. Energetically optimal stride frequency is maintained with fatigue in trained ultramarathon runners. J Sci Med Sport. (2019) 22:1054–8. 10.1016/j.jsams.2019.04.003 31029549

[4] Giovanelli N Taboga P Lazzer S. Changes in running mechanics during a 6-hour running race. Int J Sports Physiol Perform. (2017) 12:642–7. 10.1123/ijspp.2016-0135 27768506

[5] Matta GG Bossi AH Millet GY Lima P Lima JP Hopker JG. Influence of a slow-start on overall performance and running kinematics during 6-h ultramarathon races. Eur J Sport Sci. (2020) 20:347–56. 10.1080/17461391.2019.1627422 31154905

[6] Rogers B Mourot L Doucende G Gronwald T. Fractal correlation properties of heart rate variability as a biomarker of endurance exercise fatigue in ultramarathon runners. Physiol Rep. (2021) 9:e14956. 10.14814/phy2.14956 34291602 PMC8295593

[7] Wollseiffen P Schneider S Martin LA Kerhervé HA Klein T Solomon C. The effect of 6 h of running on brain activity, mood, and cognitive performance. Exp Brain Res. (2016) 234:1829–36. 10.1007/s00221-016-4587-7 26892883

[8] Smith DL Haworth JL Brooks EK Cousins JM. Postural control, dual task performance and executive function following an ultramarathon. Percept Mot Skills. (2021) 128:2767–86. 10.1177/00315125211044351 34474623

[9] Knechtle B Weiss K Villiger E Scheer V Gomes TN Gajda R The sex difference in 6-h ultra-marathon running-the worldwide trends from 1982 to 2020. Medicina (B Aires). (2022) 58:179. 10.3390/medicina58020179 PMC8876730 35208503

[10] Ehrensperger L Knechtle B Rüst CA Rosemann T. Participation and performance trends in 6-hour ultra-marathoners: a retrospective data analysis of worldwide participation from 1991 to 2010. JHSE. (2013) 8:905–24. 10.4100/jhse.2013.84.03

[11] Knechtle B Scheer V Nikolaidis PT Sousa CV. Participation and performance trends in the oldest 100-km ultramarathon in the world. Int J Environ Res Public Health. (2020) 17:1719. 10.3390/ijerph17051719 32155703 PMC7084458

[12] León-Guereño P Galindo-Domínguez H Balerdi-Eizmendi E Rozmiarek M Malchrowicz-Mośko E. Motivation behind running among older adult runners. BMC Sports Sci Med Rehabil. (2021) 13:138. 10.1186/s13102-021-00366-1 34715913 PMC8555191

[13] Thuany M Gomes TN Villiger E Weiss K Scheer V Nikolaidis PT Trends in participation, sex differences and age of peak performance in time-limited ultramarathon events: a secular analysis. Medicina (B Aires). (2022) 58:366. 10.3390/medicina58030366 PMC8952003 35334541

[14] Knechtle B Rosemann T Nikolaidis P. The role of nationality in ultra-endurance sports: the paradigm of cross-country skiing and long-distance running. Int J Environ Res Public Health. (2020) 17:2543. 10.3390/ijerph17072543 32276349 PMC7177835

[15] Thuany M Valero D Villiger E Forte P Weiss K Nikolaidis PT A machine learning approach to finding the fastest race course for professional athletes competing in ironman(®) 70.3 races between 2004 and 2020. Int J Environ Res Public Health. (2023) 20:3619. 10.3390/ijerph20043619 36834311 PMC9963404

[16] Sehovic E Knechtle B Rüst CA Rosemann T. 12-hour ultra-marathons—increasing worldwide participation and dominance of europeans. J Hum Sport Exerc. (2013) 8(4):932–53. 10.4100/jhse.2013.84.05

[17] Scheer V Di Gangi S Villiger E Rosemann T Nikolaidis PT Knechtle B. Participation and performance analysis in children and adolescents competing in time-limited ultra-endurance running events. Int J Environ Res Public Health. (2020) 17:1628. 10.3390/ijerph17051628 32138338 PMC7084740

[18] Thuany M Weiss K Villiger E Scheer V Ouerghi N Gomes TN A macro to micro analysis to understand performance in 100-mile ultra-marathons worldwide. Sci Rep. (2023) 13:1415. 10.1038/s41598-023-28398-2 36697457 PMC9876921

[19] Vitti A Nikolaidis PT Villiger E Onywera V Knechtle B. The “New York city marathon”: participation and performance trends of 1.2M runners during half-century. Res Sports Med. (2020) 28:121–37. 10.1080/15438627.2019.1586705 30889965

[20] Besson T Macchi R Rossi J Morio CYM Kunimasa Y Nicol C Sex differences in endurance running. Sports Med. (2022) 52(6):1235–57. 10.1007/s40279-022-01651-w 35122632

[21] Knechtle B Witthöft A Valero D Thuany M Nikolaidis PT Scheer V Elderly female ultra-marathoners reduced the gap to male ultra-marathoners in Swiss running races. Sci Rep. (2023) 13:12521. 10.1038/s41598-023-39690-6 37532766 PMC10397271

[22] Ivan S Daniela O Jaroslava BD. Sex differences matter: males and females are equal but not the same. Physiol Behav. (2023) 259:114038. 10.1016/j.physbeh.2022.114038 36423797

[23] Capranica L Piacentini MF Halson S Myburgh KH Ogasawara E Millard-Stafford M. The gender gap in sport performance: equity influences equality. Int J Sports Physiol Perform. (2013) 8:99–103. 10.1123/ijspp.8.1.99 23302143

[24] Senne J. Examination of gender equity and female participation in sport. Sport J. (2016) 22:1–9.

[25] Nikolaidis PT Knechtle B. Age of peak performance in 50-km ultramarathoners—is it older than in marathoners? Open Access J Sports Med. (2018) 9:37–45. 10.2147/oajsm.S154816 29535560 PMC5840300

[26] Nikolaidis PT Rosemann T Knechtle B. Force-velocity characteristics, muscle strength, and flexibility in female recreational marathon runners. Front Physiol. (2018) 9:1563. 10.3389/fphys.2018.01563 30450057 PMC6224357

[27] Balducci P Clémençon M Trama R Blache Y Hautier C. Performance factors in a mountain ultramarathon. Int J Sports Med. (2017) 38:819–26. 10.1055/s-0043-112342 28799161

[28] Knechtle B Valeri F Zingg MA Rosemann T Rüst CA. What is the age for the fastest ultra-marathon performance in time-limited races from 6 h to 10 days? Age (Dordr). (2014) 36:9715. 10.1007/s11357-014-9715-3 25280550 PMC4185021

[29] Seffrin A Vivan L Dos Anjos Souza VR da Cunha RA de Lira CAB Vancini RL Impact of aging on maximal oxygen uptake adjusted for lower limb lean mass, total body mass, and absolute values in runners. Geroscience. (2024) 46:913–21. 10.1007/s11357-023-00828-z 37233883 PMC10214322

[30] Ganse B Kleerekoper A Knobe M Hildebrand F Degens H. Longitudinal trends in master track and field performance throughout the aging process: 83,209 results from Sweden in 16 athletics disciplines. Geroscience. (2020) 42:1609–20. 10.1007/s11357-020-00275-0 33048301 PMC7732911

[31] WHO. World population ageing 2020 highlights: Living arrangements of older persons (2020).

[32] Kibler WB Putukian M. Selected issues for the master athlete and the team physician: a consensus statement. Med Sci Sports Exerc. (2010) 42:820–33. 10.1249/MSS.0b013e3181d19a0b 21099759

[33] Stambulova NB. Theoretical developments in career transition research: contributions of European sport psychology. In: Raab M Seiler R Hatzigeorgiadis A Wylleman P Elbe A-M, editors. Sport and Exercise Psychology Research: From Theory to Practice. London: Elsevier Academic Press (2016). p. 251–68. 10.1016/B978-0-12-803634-1.00012-1

[34] Bouscaren N Millet GY Racinais S. Heat stress challenges in marathon vs. ultra-endurance running. Front Sports Act Living. (2019) 1:59. 10.3389/fspor.2019.00059 33344982 PMC7739648

[35] Berger NJA Best R Best AW Lane AM Millet GY Barwood M Limits of ultra: towards an interdisciplinary understanding of ultra-endurance running performance. Sports Med. (2024) 54:73–93. 10.1007/s40279-023-01936-8 37751076

[36] Shoak MA Knechtle B Rüst CA Lepers R Rosemann T. European dominance in multistage ultramarathons: an analysis of finisher rate and performance trends from 1992 to 2010. Open Access J Sports Med. (2013) 4:9–18. 10.2147/oajsm.S39619 24379704 PMC3871050

[37] Davids K Araújo D Vilar L Renshaw I Pinder R. An ecological dynamics approach to skill acquisition: implications for development of talent in sport. Talent Dev Excell. (2013) 5:21–34.

